# The changes we need: Education post COVID-19

**DOI:** 10.1007/s10833-021-09417-3

**Published:** 2021-02-18

**Authors:** Yong Zhao, Jim Watterston

**Affiliations:** 1 Melbourne Graduate School of Education, University of Melbourne, Parkville, Australia; 2 School of Education, University of Kansas, 419 JRP, Lawrence, KS 66049 USA

## Abstract

The COVID-19 pandemic has caused both unprecendented disruptions and massive changes to education. However, as schools return, these changes may disappear. Moreover, not all of the changes are necessarily the changes we want in education. In this paper, we argue that the pandemic has created a unique opportunity for educational changes that have been proposed before COVID-19 but were never fully realized. We identify three big changes that education should make post COVID: curriculum that is developmental, personalized, and evolving; pedagogy that is student-centered, inquiry-based, authentic, and purposeful; and delivery of instruction that capitalizes on the strengths of both synchronous and asynchronous learning.

## Introduction

The impact of the COVID-19 pandemic on education is both unprecedented and widespread in education history, impacting nearly every student in the world (UNICEF 2020; United Nations 2020). The unexpected arrival of the pandemic and subsequent school closures saw massive effort to adapt and innovate by educators and education systems around the world. These changes were made very quickly as the prevailing circumstances demanded. Almost overnight, many schools and education systems began to offer education remotely (Kamanetz 2020; Sun et al. 2020). Through television and radio, the Internet, or traditional postal offices, schools shifted to teach students in very different ways. Regardless of the outcomes, remote learning became the de facto method of education provision for varying periods. Educators proactively responded and showed great support for the shifts in lesson delivery. Thus, it is clear and generally accepted that “this crisis has stimulated innovation within the education sector” (United Nations 2020, p. 2).

However, the changes or innovations that occurred in the immediate days and weeks when COVID-19 struck are not necessarily the changes education needs to make in the face of massive societal changes in a post-COVID-19 world. By and large, the changes were more about addressing the immediate and urgent need of continuing schooling, teaching online, and finding creative ways to reach students at home rather than using this opportunity to rethink education. While understandable in the short term, these changes will very likely be considered insubstantial for the long term.

The COVID-19 pandemic has the potential to be a once in a generation opportunity for real change a number of reasons. First, the pandemic was global and affected virtually all schools. As such, it provides the opportunity for educators and children to come together to rethink the education we actually need as opposed to the inflexible and outdated model that we are likely to feverishly cling to. Second, educators across the world demonstrated that they could collectively change en masse. The pandemic forced closure of schools, leaving teachers, children and adults to carry out education in entirely different situations. Governments, education systems, and schools offered remote learning and teaching without much preparation, planning, and in some cases, digital experience (Kamanetz 2020; Sun et al. 2020). Third, when schools were closed, most of the traditional regulations and exams that govern schools were also lifted or minimally implemented. Traditional accountability examinations and many other high stakes tests were cancelled. Education was given the room to rapidly adapt to the prevailing circumstances.

It is our hope that as we transition out of the COVID-19 pandemic and into an uncertain future that we can truly reimagine education. In light of this rare opportunity, we wish to urge scholars, policy makers, and educators to have the courage to make bold changes beyond simply changing instructional delivery. The changes that we advocate in this paper are not new but they never managed to gain traction in the pre-COVID-19 educational landscape. Our most recent experience, however, has exacerbated the need for us to rethink what is necessary, desirable, and even possible for future generations.

## Changes we need

It is incumbant upon all educators to use this crisis-driven opportunity to push for significant shifts in almost every aspect of education: what, how, where, who, and when. In other words, education, from curriculum to pedagogy, from teacher to learner, from learning to assessment, and from location to time, can and should radically transform. We draw on our own research and that of our colleagues to suggest what this transformation could look like.

## Curriculum: What to teach

It has been widely acknowledged that to thrive in a future globalized world, traditionally valued skills and knowledge will become less important and a new set of capabilities will become more dominant and essential (Barber et al. 2012; Florida 2012; Pink 2006; Wagner 2008; Wagner and Dintersmith 2016). While the specifics vary, the general agreement is that repetition, pattern-prediction and recognition, memorization, or any skills connected to collecting, storing, and retrieving information are in decline because of AI and related technologies (Muro et al. 2019). On the rise is a set of contemporary skills which includes creativity, curiosity, critical thinking, entrepreneurship, collaboration, communication, growth mindset, global competence, and a host of skills with different names (Duckworth and Yeager, 2015; Zhao et al. 2019).

For humans to thrive in the age of smart machines, it is essential that they do not compete with machines. Instead, they need to be more human. Being unique and equipped with social-emotional intelligence are distinct human qualities (Zhao 2018b, 2018c) that machines do not have (yet). In an AI world individual creativity, artistry and humanity will be important commodities that distinguish us from each other.

Moreover, given the rapidity of changes we are already experiencing, it is clear that lifelong careers and traditional employment pathways will not exist in the way that they have for past generations. Jobs and the way we do business will change and the change will be fast. Thus there are almost no knowledge or skills that can be guaranteed to meet the needs of the unknown, uncertain, and constantly changing future. For this reason, schools can no longer preimpose all that is needed for the future before students graduate and enter the world.

While helping students develop basic practical skills is still needed, education should also be about development of humanity in citizens of local, national, and global societies. Education must be seen as a pathway to attaining lifelong learning, satisfaction, happiness, wellbeing, opportunity and contribution to humanity. Schools therefore need to provide comprehensive access and deep exposure to all learning areas across all years in order to enable all students to make informed choices and develop their passions and unique talents.

A new curriculum that responds to these needs must do a number of things. First, it needs to help students develop the new competencies for the new age (Barber et al. 2012; Wagner 2008, 2012; Wagner and Dintersmith 2016). To help students thrive in the age of smart machines and a globalized world, education must teach students to be creative, entrepreneurial, and globally competent (Zhao 2012a, 2012b). The curriculum needs to focus more on developing students’ capabilities instead of focusing only on ‘template’ content and knowledge. It needs to be concerned with students’ social and emotional wellbeing as well. Moreover, it needs to make sure that students have an education experience that is globally connected and environmentally connected. As important is the gradual disappearance of school subjects such as history and physics for all students. The content is still important, but it should be incorporated into competency-based curriculum.

Second, the new curriculum should allow personalization by students (Basham et al. 2016; Zhao 2012b, 2018c; Zhao and Tavangar 2016). Although personalized learning has been used quite elusively in the literature, the predominant model of personalized learning has been computer-based programs that aim to adapt to students’ needs (Pane et al. 2015). This model has shown promising results but true personalization comes from students’ ability to develop their unique learning pathways (Zhao 2018c; Zhao and Tavangar 2016). That is, students can follow their passions and strengths. This not only requires the curriculum to be flexible so that students can choose what they wish to learn, but also requires students to come up with their own learning pathway without being overly constrained by the pre-determined curriculum. Thus national curriculum or curriculum for all students should be a minimal suite of essential knowledge and skills, sufficient for all students to develop the most basic competences and learn the most common norms, expectations, and the societal organizations of a jurisdiction.

Enabling students to co-develop part of the curriculum is not only necessary for them to become unique but also gives them the opportunity to exercise their right to self-determination, which is inalienable to all humans (Wehmeyer and Zhao 2020). It provides the opportunities for students to make choices, propose new learning content, and learn about consequences of their actions. Furthermore, it helps students to become owners of their learning and also develop life-long learning habits and skills. It is to help them go meta about their learning—above what they learn and understand why they learn.

Third, it is important to consider the curriculum as evolving. Although system-level curriculum frameworks have to be developed, they must accommodate changes with time and contexts. Any system-level curriculum should enable the capacity for schools to contextualize and make changes to it as deemed necessary. Such changes must be justifiable of course but a system-level curriculum framework should not use national or state level accountability assessments to constrain the changes.

## Pedagogy: How to teach

There is increasing call for learners to be more actively engaged in their own learning. The reasons for students to take a more significant role in their own learning are multiple. First, students are diverse and have different levels of abilities and interests that may not align well with the content they are collectively supposed to learn in the classroom. Teachers have been encouraged to pursue classroom differentiation (Tomlinson 2014) and students have been encouraged to play a more active role in defining their learning and learning environments in collaboration with teachers (Zhao 2018c). Second, the recent movement toward personalized learning (Kallick and Zmuda 2017; Kallio and Halverson 2020) needs students to become more active in understanding and charting their learning pathways.

To promote student self-determination as both a self-evident, naturally born right and an effective strategy for enhanced learning (Wehmeyer and Zhao 2020), we need to consider enabling students to make informed decisions regarding their own learning pathway. This generation of learners are much more active and tech-savvy. They access information instantly and have been doing so throughout their daily life. They have different strengths and weaknesses. They also have different passions. Thus, schools should use discretion to start relaxing the intense requirements of curriculum. Schools could start by allowing students to negotiate part of their curriculum instead of requiring all students learn the same content, as discussed earlier. Students should be enabled to have certain levels of autonomy over what they want to learn, how they learn, where they learn and how they want to be assessed (Zhao 2018c). When students have such autonomy, they are more likely to be less constrained by the local contexts they are born into. The impact of their home background and local schools may be less powerful.

Students should exercise self-determination as members of the school community (Zhao 2018c). The entire school is composed of adults and students, but students are the reason of existence for schools. Thus, schools and everything in the school environment should incorporate and serve the students, yet most schools do not have policies and processes that enable students to participate in making decisions about the school—the environment, the rules and regulations, the curriculum, the assessment, and the adults in the school. Schools need to create these conditions through empowering students to have a genuine voice in part of how they operate, if not in its entirety. Students’ right to self-determination implies that they have the right to determine under what conditions they wish to learn. Thus, it is not unreasonable for schools to treat students as partners of learning and of change (Zhao 2011, 2018c).

It should not be unique to see school practices co-developed with students (Zhao 2018c). Students not only will be co-owners (with parents and teachers) of their own learning enterprise, but also co-owners of the school community. It is likely to see students having their own personal learning programs and also acting as fully functioning members of the entire school community, contributing to fundamental decisions regarding the curriculum for all, the staff, the students, and the entire environment.

Moreover, with ubiquitous access to online resources and experts, students do not necessarily need teachers to continually and directly teach them. When students are enabled to own their learning and have access to resources and experts, the role of the teacher changes (Zhao 2018a). Teachers no longer need to serve as the instructor, the sole commander of information to teach the students content and skills. Instead, the teacher serves other more important roles such as organizer of learning, curator of learning resources, counselor to students, community organizer, motivator and project managers of students’ learning. The teacher’s primary responsibility is no longer simply just instruction, which requires teacher education to change as well. Teacher education needs to focus more on preparing teachers to be human educators who care more about the individual students and serve as consultants and resource curators instead of teaching machines (Zhao 2018a).

Pedagogy should change as well. Direct instruction should be cast away for its “unproductive successes” or short-term successes but long term damages (Bonawitza et al. 2011; Buchsbauma et al. 2011; Kapur 2014, 2016; Zhao 2018d). In its place should be new models of teaching and learning. The new models can have different formats and names but they should be student-centered, inquiry-based, authentic, and purposeful. New forms of pedagogy should focus on student-initiated explorations of solutions to authentic and significant problems. They should help students develop abilities to handle the unknown and uncertain instead of requiring memorization of known solutions to known problems.

## Organization: Where and when to teach

Technology has made it possible for schools to offer online education for quite some time and the number of students taking online courses has been on the rise, but not until the arrival of COVID-19 has the majority of education been offered through this mode. While there are many good reasons for schools to return to what was refrred to as “normal,” the normalcy may not be easily achieved because of the uncertainty of the virus, and as discussed above, may not even be desirable.

Moving teaching online is significant. It ultimately changed one of the most important unwritten school rules: all students must be in one location for education to take place. The typical place of learning has been the classroom in a school and the learning time has been typically confined to classes. This massive online movement changed the typical. It has forced teachers to experience remote teaching without proximity to the students. It has also given many teachers the opportunity to rethink the purpose of teaching and connecting with students.

When students are not learning in classes inside a school, they are distributed in the community. They can interact with others through technologies. This can have significant impact on learning activities. If allowed or enabled by a teacher, students could be learning from online resources and experts anywhere in the world. Thus, the where of learning changes from the classroom to the world.

Furthermore, the time of learning also changes. When learning goes online and students are not or do not need to be in schools, their learning time vastly expands beyond the traditional school time. They can learn asynchronously at anytime. Equally important is that their learning time does not need to be synchronous with each other or with that of the teacher.

There are many possible ways for schools to deliver remote learning (Zhao 2020). The simplest is to simulate that schools are open with traditional timetables with the default model being that all students attend lessons on screen at the same time as they do in schools. In this case, nothing changes except for the fact that students are not in the same location as their classmates and the teacher. While it has been perhaps the most common approach that has been taken by many schools, this approach has not been very effective and successful, resulting in distress, disengagement, and much less personal interaction and learning than traditional face-to-face situations (Darby 2020; Dorn et al. 2020).

As schools continue to explore online learning, new and more effective models are being explored, innovatively developed, and practiced. The more effective models of online learning have a well-balanced combination of both synchronous and asynchronous sessions that enable more desirable ways of learning. Instead of teaching online all the time, it is possible, for example, to conduct inquiry-based learning. Students receive instructions from online resources or synchronous meetings, conduct inquiry, create products individually or within small groups, and make presentations in large class synchronous meetings. Instead of lecturing to all students, teachers could create videos of lectures or find videos made by others and share them with students. They would also be meeting with small groups of individuals for specific advice and support. The fundamental pursuit is that there is minimal benefit or student engagement for teachers to lecture all the time when more interesting and challenging instructional models can be developed.

Today, being disconnected physically can result in being more broadly connected virtually. Students have been traditionally associated with their schools and schools have typically served local communities. Thus, students typically are connected and socialize with their peers from restricted catchment areas. Despite the possibility to connect globally with people from other lands, most schools’ activities are local. Today, when local connections become less reliable and students are encouraged to have social distancing, it is possible to encourage more global connections virtually. Students could join different learning communities that involve members from different locations, not necessarily from their own schools. Students could also participate in learning opportunities provided by other providers in remote locations. Furthermore, students could create their own learning opportunities by inviting peers and teachers from other locations.

The ideal model of organizing students, based on the COVID-19 experiences, is perhaps a combination of both online and face-to-face learning opportunities. Many schools have already reopened, but when schools reopen it is unnecessary to undo the online aspect of learning developed during COVID-19. Online learning can be effective (Means et al. 2013; Rudestam and Schoenholtz-Read 2010; Zhao et al. 2005), but a well-designed mixed mode delivery of online and face-to-face education should be more effective for learning in general but especially so should there be future instances of virtual learning (Tucker 2020). The idea of blended learning or flipped classrooms (Bishop and Verleger 2013) has been promoted and researched in recent years as very effective models of teaching. COVID-19 should have made the convincing much easier since many teachers have been forced to move online.

When learning is both online and face-to-face, students are liberated from having to attend classes at specific times. They are also no longer required to be in the same place to receive instruction from teachers. They could work on their own projects and reach out to their teachers or peers when necessary. When students are no longer required to attend class at the same time in the same place, they can have much more autonomy over their own learning. Their learning time expands beyond school time and their learning places can be global.

## Summary

Education will undoubtedly go through major changes in the next decade as the combined result of multiple major forces. These changes include curricular changes that determine what is to be learned by learners. It is likely that more students will be moving toward competency-based learning that has an emphasis on developing unique skills and abilities. Learning has to become more based on strengths and passions and become personalized. In response, education providers will need to make student autonomy and student agency key to transforming pedagogy and school organizations. Students will prosper by having more say in their own learning and their learning communities. Moreover, schools will have a unique opportunity to positively and proactively change as a result of COVID-19 and the need for global connections. It is possible to see schools rearrange their schedules and places of teaching so that students can at the same time take part in different and more challenging learning opportunities regardless of their physical locations. Relevant online learning will be on the rise and perhaps becomes a regular part of the daily routine for many students.

Of course, we cannot forget that not all students have equal access to technology, both in terms of hardware and digital competency. The issue of digital divide remains a significant issue around the globe. It is important for us to reimagine a better education with technology and find creative ways to make education more equitable, including wiping out the digital divide.
